# Moral Hazard and the Demand for Dental Treatment: Evidence from a Nationally Representative Survey in Thailand

**DOI:** 10.1155/2022/2259038

**Published:** 2022-08-17

**Authors:** Krichkanok Srimuang, Piriya Pholphirul

**Affiliations:** ^1^Graduate School of Development Economics, National Institute of Development Administration, Serithai Road, Klong-Chan, Bangkapi, Bangkok 10240, Thailand; ^2^Center of Development Economics Studies, National Institute of Development Administration, Serithai Road, Klong-Chan, Bangkapi, Bangkok 10240, Thailand

## Abstract

Even though there are examples in the health economics literature of evidence investigating moral hazard in health insurance provided by general healthcare services, studies of moral hazard in dental care in developing countries are still scarce, especially when it comes to nationally representative data on dental care demand and expenditure. Using Thailand as a case study, we investigate here whether moral hazard in dental insurance exists and, if so, the extent to which it affects different dental insurance on the demand for dental care in developing countries. We use a nationally representative sample of 269,206 individuals to quantify the impacts of dental care insurance on four sets of dependent variables describing demand for dental care. They are: (i) dental care utilization, (ii) numbers of dental care visits, (iii) dental care expenditure, and (iv) each type of dental care. Our probit and tobit estimations show that there is no evidence of the existence of moral hazard in terms of dental care utilization and dental care expenditure. However, there is a moral hazard of dental insurance existence on the number of dental care visits and type of dental care. People with generous dental benefits coverage tend to use preventive dental treatments along with necessary treatment but also use costly restorative dental treatments more than do those with lower coverage. It can thus be concluded that, in the case of developing countries, dental care insurance is found to increase the use of dental care, especially for preventive care.

## 1. Introduction

In general, health insurance induces consumers to use healthcare services. But undesirable behavior may occur when insurance coverage leads to unhealthy outcomes and an increase in the cost of health care. These issues relate to the issue of moral hazard in health insurance, which is divided into two main categories. First, in the case of ex ante moral hazard, consumers reduce measures to maintain their health or increase unhealthy behaviour, for instance, exercising less, or smoking and drinking more because they have health insurance coverage (see [1, 2]. Second is ex post moral hazard; when the cost of medical care is determined not only by the type of illness or disease a patient experiences but also by the patient's willingness to use medical services (from a demand side perspective) as well as the decision of a physician/healthcare provider (from a supply side perspective) [3, 4].

Empirical studies provide evidence of moral hazard in health insurance according to conventional ex post moral hazard theory, as noted previously. Einav and Finkelstein [5] demonstrate that health insurance increases healthcare utilization and spending across the board, including hospital admissions, emergency department visits, primary care, preventive care, and prescription drugs. Lowering consumer cost-sharing, therefore, leads to more healthcare spending.

Besides the case of general healthcare, the study of moral hazard can also be extended to dental care, which relates to dental insurance and the relatively expensive cost of dental treatment [6]. This may, in turn, lead to moral hazard in dental insurance. The effect of dental insurance on the demand for dental care has been studied in a randomized trial of health insurance focusing on the number of dental visits and expenditure on dental services of insured people between 1974 and 1982 in the United States. Among the insured, those with insurance policies with lower cost-sharing or generous coverage tended to avail themselves of dental services to a greater extent. For example, participants on free plans had 34 percent more dental visits and 46 percent higher dental expenses than those on a 95 percent plan. Although the study did not examine people without dental coverage, it is expected that they would have sought more dental care if they had enrolled in at least a 95 percent plan [7]. Another study demonstrated the interrelationships between dental health, private dental insurance, and the use of dental services in Australia, where the dental healthcare system is dominated by private fee-for-service provisions. A sample of 11,231 adults aged 15 years and over from the Australian National Survey of Adult Oral Health found that having private supplementary insurance was associated with a 56 percent higher probability of seeing a dentist [8].

The impact of having insurance on the demand for the main types of dental services has also been studied in the US. Each type of dental service was classified in terms of preventive services (exams, cleanings, sealants, and x-rays), basic restorative services (fillings, periodontics, extractions, and periodontics), or major restorative services (crowns, root canals, and dentures) because these dental services have different cost-sharing and demand characteristics. The study used data from the 2001–2006 Medical Expenditure Panel Survey, which used a sample size of 53,133 Americans, both privately insured and uninsured. A probit model estimation indicated that dental coverage increased the probability of preventive care by 19 percent and that of restorative services by 11 to 16 percent [9].

Even though the literature on the moral hazard of dental care mentioned above measures usage in terms of dental care expenditure and the number of dental visits, no research has yet been undertaken to investigate the impacts on the types of dental treatment. In addition, dental insurance can come with different types of schemes in some developing countries, which can result in different rates of dental care utilization. Even though developing countries tend to lack nationally representative data, there have nevertheless been empirically compelling studies to demonstrate the demand for dental care and its determinants, especially with regard to dental insurance. For these reasons, Thailand is a good example of a developing country where individuals are covered by different dental insurance schemes, in the Thai case, by the Universal Coverage Scheme (UCS), the Social Security Scheme (SSS), and the civil servant medical benefits scheme (CSMBS). These three schemes cover similar comprehensive dental services such as oral examinations, scaling, fillings, extractions, and wisdom tooth removal. Nevertheless, the dental benefits under each scheme have different coverage details in terms of cost coverage and additional services, as shown in Table 1.

Comparing the three schemes, we see that removable partial and complete dentures were included in all three schemes. However, other services were included in both CSMBS and UCS but not in SSS, for instance, root canal treatment on primary teeth and orthodontics (only for cleft lip and palate patients and those who had suffered accidents, but not merely for cosmetic purposes). Some dental services were included under either CSMBS or UCS. For example, CSMBS covered root canal treatment on permanent teeth whereas UCS covered sealant treatment for children and adolescents plus fluoride treatment for school-age children and persons with high-risk caries. These preventive treatments in UCS applied to all Thai targeted citizens regardless of their schemes.

Our study here, therefore, investigates further whether moral hazard in different dental insurance schemes exists and whether different dental insurance affects demand for dental care in Thailand. Health and Welfare Surveys were conducted by Thailand's National Statistical Office in the years 2015, 2017, and 2019 to investigate the impacts of different insurance schemes on the demand for dental care. This demand for dental care was measured not only in terms of the probability of dental care utilization, the number of dental visits, and dental care expenditure, but also by the use of each type of dental care service, for instance, (1) preventive care (oral examination, scaling, and sealant), (2) basic restorative care (filling and extraction), and (3) major restorative care (root canal treatment, dentures, and orthodontics).

This research article consists of five sections.  Section 2 presents a literature review on the moral hazard in dental care insurance and the demand for dental care in the case mainly in developing countries.  Section 3 explains the data set and descriptive statistics.  Section 4 conducts an econometrics estimation that examines whether moral hazard in dental care insurance exists and to what extent dental care is utilized by Thai people. And, Section 5 provides the conclusions and policy recommendations.

## 2. Literature Review on Moral Hazard and Demand for Dental Care

Oral health and disease are strongly age-related. Both children with complete primary teeth and adolescents, until they become adults with permanent teeth, should be monitored for caries prevalence and periodontal disease because these are the major oral diseases in all population groups [12]. But also to note: different socioeconomic gradients are also associated with oral health status and dental disease. Thus, individuals with lower incomes and level of education frequently tend to have poorer self-reported oral health and higher cases of untreated dental caries [13]. Their extraneous circumstances often determine whether they visit their dentists or put off such visits and regular care as well as and what type of dental care that is available to them.

Therefore, the effects of demographic and socioeconomic characteristics along with demand for dental care have been investigated extensively in many developed countries, where data sets tend to be available. The important determinants on demand have been examined repeatedly, and each has been found to have a similar effect. For example, individuals with higher incomes and education are shown to have a higher probability of dental visits [14, 15]. Those who are female and married tend to be more likely to have utilized dental care [16]. Although the probability of dental care utilization declines over a person's lifetime, and older people tend to visit dentists less frequently, the annual frequency of dental visits actually increases with age until middle age. After age 65, visits stabilize at a mean of one visit annually [14]. And, individuals residing in urban areas tend to have higher rates of any type of preventive or curative treatment compared to those residing in rural areas [17]. Currently, teledentistry has been implemented to provide dental care-especially for diagnosis and consultation, especially in the context of COVID-19 and in remote regions. This increases access for patients to get the initial dental care they need [18]. Finally, some occupations have relatively lower dental care utilization rates, such as forestry and fishing, construction, farm and other agricultural work, and food service, all of which are also associated with a high poverty rate [19].

Therefore, dental insurance is positively associated with the demand for dental care. For example, Bhatti et al. [14]; using a sample from the Canadian Community Health Survey (CCHS) of 108,861 respondents aged 25 years or above, found that the probability of receiving dental care increased by 17 percent for those having dental insurance compared to the uninsured.

In the United States, many empirical measures have been used to assess the impact of insurance on the demand for dental care. Manski et al. [15], for example, found that people with private coverage had more demand for dental care than did those without coverage, as measured by the probability of visiting a dentist, the number of dental visits, and the expenditure, other factors kept constant. In a study of 8,542 white adults who were not eligible for Medicaid from the data of the 1977 National Medical Care Expenditure Survey (NMCES), Mueller and Monheit [20] compared dental care utilization between the uninsured, those insured with coinsurance—at lower or higher rates (first-dollar coverage), and those with a deductible plan. The results suggested that the primary effects of dental insurance were to facilitate access to care and to increase dental expenditure. Dental benefits coverage for low-income people and dental care utilization were also examined. For example, Medicaid adult dental coverage increased the likelihood that people had visited the dentist in the previous six months or year. The magnitude of this effect varied with the level of the Medicaid-to-private fee ratio for dentists. For instance, Medicaid adult dental coverage was associated with an increase of 12.9 percent in the probability of a yearly dental visit [21]. The expansion benefits of Medicaid were also associated with an increase in complete teeth loss because people with poor oral health may have accessed dental services, especially for tooth extractions [22].

Sweet et al. [23] found that privately insured enrollees (Delta Dental Plan of Iowa) were more likely to use dental services than were those covered by Medicaid. However, Medicaid members were nearly twice as likely as private insurers to receive endodontic treatment and nearly four times as likely to have had a tooth extracted. The greater degree of tooth extractions on the part of Medicaid patients reflected their lower oral health status, even though Medicaid covered a much younger group of adults.

In the United Kingdom, there have been clear socioeconomic variations in the utilization of preventive and restorative services. People with manual occupations and the least educated have been consistently less likely to have preventive or restorative dental services than were those at the top of occupational classifications and the highly educated. Conversely, the same groups at the bottom of the social hierarchy were more likely to ever have had tooth extractions than were those at the top. This suggests that individuals who were the least educated and worked at-low paying jobs tended to have more definitive dental treatment such as extractions instead of treatments that require appointments and repeated visits to the dentists [24]. A current study in Japan showed that average income per head and college enrollment were positively associated with preventive care and were negatively associated with curative care. The findings suggest that there may be fewer dental visits for preventive care since people with lower income and education levels tend to have dental diseases that had become serious by the time they visited the dentist [25].

A cross-sectional study in western Iran's Kermanshah province discovered that of a total of 894 household heads, only 18.2 percent had dental insurance. The results showed that those who were older, had a higher income, a higher level of education, and who rated themselves as having poor oral health and who did not regularly brush their teeth were the ones who tended to utilize dental services. Moreover, people with dental insurance had a higher probability of dental care utilization and a greater frequency of visits to dentists than did those without dental insurance. Having insurance increased dental care accessibility by reducing the cost of dental services since private sectors were the main providers (80 percent) of dental care services in Iran [26]. In another cross-sectional study in Iran, based on telephone interviews, 58 percent of a total of 6,029 adult participants had public insurance, and 28 percent had both public and commercial insurance. This indicates that people with both public and commercial insurance coverage were more likely to visit dentists and undergo dental check-ups [27].

Another empirical study in Chile has implemented universal insurance via full or partial public subsidies. It is found that the use of dental care significantly increased between 2004 and 2009, especially among those with public health insurance, with a lower educational level, and of lower socioeconomic status [28].

## 3. Data

To investigate whether moral hazard in dental insurance exists and to assess the impact of different dental insurance plans on the demand for dental care in Thailand, this study used secondary data from three data sets, from the 2015, 2017, and 2019 Health and Welfare Surveys in Thailand, conducted by the Thailand National Statistical Office. The data sets comprised nationally representative surveys, which covered approximately 269,206 Thai people and all age groups.

As shown in Table 2, percent of respondents were female and 47.5 percent male. Age groups of respondents were 0–15 years (19.2 percent), 16–24 years (9.4 percent), 25–34 years (11.1 percent), 35–44 years (14.6 percent), 45–54 years (17.2 percent), 55–64 years (14.8 percent), and 65 years and over (13.7 percent). These age groups were classified according to oral health conditions and care needs in each age group [12]. As for education, those with no formal education accounted for 4.9 percent, preschool 21.5 percent, primary school 34.4 percent, secondary school 13.7 percent, high school 12.3 percent, and the remaining respondents had received at least a university diploma. The majority (62 percent) were married, 23.9 percent were single, and 14.1 percent were widowed or divorced or separated. In terms of occupation, the respondents were unemployed (30.7 percent), farmers (26.8 percent), services or sales workers (14 percent), unskilled workers (7.5 percent), crafts workers (6.7 percent), or factory workers (4.6 percent). The rest were public servants, professionals, technicians, and clerks. Average monthly income per head was categorized according to quintiles. The mean of the first quintile (Q1) was 773 baht (21.6 percent) and the mean of Q5 was 32,375 baht (19.7 percent). As for dental insurance, there were insured and uninsured persons, 99.6 and 0.4 percent, respectively. Among the insured, there were three main types of public dental insurance: UCS (44.4 percent), SSS (10.9 percent), and CSMBS (11.5 percent).

The number of dental care visits in the previous 12 months is shown in Table 2 by tabulation, indicating that most respondents had not visited a dentist in the previous 12 months (0 visits). The proportion of people who did not use dental services was approximately 92–93 percent. Conversely, there were a few respondents who visited a dentist at least once in the previous 12 months, approximately 7–8 percent.

Those who visited a dentist at least once in the previous 12 months had to pay on average 533–616 baht out of pocket per person. However, when dental care expenditure was separated into deciles, the majority of patients (50.3–54.8 percent) did not have to pay any out of pocket costs (0 baht) because their dental care insurance provided full coverage. As also shown in Table 2, the first decile (D1) was 0 baht. For the sixth to tenth deciles (D6-D10), the patients had to pay on average 30 baht, 295 baht, 555 baht, 1,000 baht, and 3,624 baht, respectively.

The respondents who had completed a higher level of education and those who earned a higher average monthly income paid relatively more out of pocket costs for dental care over the previous 12 months. In terms of occupation, public servants paid on average 878 baht out of pocket for dental care, which was higher than those in other occupations paid. The majority (51.2–60.3 percent) of service or sales workers, the unemployed, and clerks also bore some out-of-pocket costs for dental care utilization in the previous 12 months, their spending on average coming to 791 baht, 728 baht, and 689 baht, respectively.

As also shown in Table 2, respondents who lived in Bangkok, the capital of Thailand, had to pay on average 1,223 baht out of pocket for dental care, which was relatively more expensive than it was for those who lived in other regions of Thailand. As for dental insurance, not surprisingly, uninsured persons had to pay on average 1,646 baht out of pocket for dental care services, which was higher than it was for those insured under UCS, SSS, and CSMBS, who spent on average only 30–252 baht. Moreover, the majority of those who used UCS (79.5 percent), SSS (67.1 percent), and CSMBS (92.9 percent) did not have to pay anything at all (free plan/0 baht) for dental care in the previous 12 months. However, even if people had dental insurance but did not use it at the time of their dental visits (insured paid by cash), they had to pay on average 1,502 baht.

Considering the utilization of each type of dental treatment as shown in Table 3 by tabulation, females tended to utilize all preventive treatments as well as basic or major treatments more than males did. Among age groups, children aged 0–15 years were more likely to undergo oral exams and get sealants and fluoride treatments compared to other age groups because of dental care programs in schools and UCS full coverage [29, 30]. Older age groups were more likely to use restorative treatments such as extraction, root canal, and denture treatments than were other age groups because of the fact that the elderly have a higher probability of losing permanent teeth.

Those with higher levels of education tended to avail themselves of preventive treatments (scaling and major restorative procedures such as orthodontics, for instance) more than did those with lower levels of education. Conversely, those with lower levels of education tended to undergo restorative services such as extractions and fillings more than did those with higher levels of education. Married persons were more likely to use both preventive and restorative treatments, except for fluoride treatments and orthodontics, than were those of other marital status. Single people tended to undergo orthodontic procedures more than did others.

As for occupation, most respondents were concentrated in the unemployed category (30.5 percent), farmers (20 percent), and service or sales workers. As a result, these occupations seemed to use both preventive and restorative treatments more than did those in other occupations. Respondents who had a higher monthly income tended to use more preventive treatments such as exams and scaling, including expensive restorative procedures such as root canals. Conversely, those who had a lower monthly income tended to get more extractions.

Regarding dental insurance, as shown in Table 3 by tabulation, people with UCS tended to use some preventive treatments such as exams and fluoride treatments, including basic restorative services such as fillings and extractions more than did those with other types of insurance and the uninsured. Because these treatments were fully covered by UCS; however, UCS beneficiaries were the majority (48 percent) of all insured. Moreover, people with any kind of dental insurance but who did not use their coverage (insured but paid by cash) tended to use major restorative care such as root canals, dentures, and orthodontic treatments more than did others in the previous 12 months. This may be because those with either UCS, SSS, or CSMBS preferred the more convenient service available from private providers and were willing to pay for it themselves. Another possibility is that coverage for some treatments, such as orthodontics, was inadequate for most people.

This analysis of moral hazard and demand for dental care in Thailand presents the descriptive statistics for the extent of dental care utilization in the previous 12 months, dental care expenditure, and each type of dental service used by the tabulation but not controlled for socioeconomic factors. Therefore, to control for individual bias and socioeconomic characteristics, this study further investigated the impact of dental insurance on demand for dental care and whether a moral hazard exists through an econometrics estimation, as presented in the next step.

## 4. Econometrics Estimation and Results

The current study employs an econometrics model to quantitatively investigate whether (desirable and undesirable) moral hazard in dental insurance exists and whether different-level coverage of dental insurance influences demand for dental care in Thailand, which serves as a case study for developing countries. The independent variables include socioeconomic characteristics comprising gender, age, religion, nationality, level of education, marital status, occupation, average monthly income per head, municipal area and region, and place of the dental visit, as well as different types of dental insurance.

For dental insurance, most Thai people (99 percent) are covered by one of the three main public health insurance schemes, which include dental care benefits. These are the universal coverage scheme (UCS), the social security scheme (SSS), and the civil servant medical benefits scheme (CSMBS). However, this study also examines other dental insurance, for instance, private insurance, insurance paid by the employer, and insurance purchased by individuals who have either UCS, SSS, or CSMBS but who did not use it when they visited the dentist and thus paid out-of-pocket (insured and paid by cash); the uninsured are also considered. Overall, UCS, SSS, and CSMBS comprise publicly funded healthcare, which offers full coverage and the free plan for comprehensive dental care. Among those three insurance options, SSS provides relatively lower coverage, which does not cover treatments such as fluoride and root canals, as shown in Table 1.

The dependent variables are as follows.

First, for dental care utilization in the previous 12 months, “respondents used any dental services = 1” or “respondents did not use any dental services = 0.” This variable is a binary dependent variable.

Second, for the number of dental visits in the past 12 months, the answers are an integer that starts from 0 (zero). For example, if the number of dental visits is 0, it means a respondent had no dental visits in the previous 12 months. If it equals 1, it means a respondent made 1 dental visit in the previous 12 months. This variable is a continuous dependent variable but it is right-skewed distribution because the majority of respondents (92–93 percent) had made no dental visits in the previous 12 months as indicated in Table 2.

The third is dental care expenditure per person at the time of visiting a dentist in the previous 12 months. This expenditure is an out-of-pocket payment by the respondent for any dental care service. The out-of-pocket cost begins from 0 baht (free plan; measured in Thai currency: baht). This variable is a continuous dependent variable, which is right-skewed distribution. The majority of respondents, who are insured and covered by the free plan, are also shown in Table 2.

Finally is each type of dental care service used in the previous 12 months. There are nine types of dental treatments in this study. They are categorized, according to Meyerhoefer et al. [9]; into three main types: preventive care, basic restorative care, and major restorative care. Oral exams, scalings, sealants, and fluoride treatments are preventive treatments. Fillings and extraction are basic restorative treatments while root canals, dentures, and orthodontics are major restorative treatments. Nevertheless, the model estimation treats each type of dental care service as a binary dependent variable, for instance, “oral exam used in the previous 12 months = 1” or “otherwise = 0,” “scaling = 1” or “otherwise = 0,” “extraction = 1” or “otherwise = 0.”

The model estimations in this study are therefore based on a probit or tobit model due to the kinds of dependent variables. A probit model is used in cases where there are two outcomes of a binary dependent variable. After running the probit model, marginal effects are estimated in order to interpret the “probability of dental care utilization” and the “probability of use of each type of dental care treatment.” A tobit model is used in cases where the continuous dependent variable is skewed to one direction and the value of the variable is not negative. This model is adopted to estimate “the number of dental visits” and the “dental care expenditure” of the Thai population.

Table 4 consists of twelve models. Model 1 shows the probability of dental care utilization in the previous 12 months. Model 2 shows the number of dental visits. Model 3 shows the dental care expenditure (baht per person). Models 4–12 demonstrate the probability of undergoing dental care treatments, which comprise oral exams, scalings, sealants, fluoride treatments, fillings, extraction, root canals, dentures, and orthodontics. All models include socioeconomic characteristics of individuals, for instance, gender, age, religion, nationality, education, marital status, occupation, average monthly income, region, and place of dental visits. The socioeconomic factors are treated as controlled variables.

Estimation results show that females have a higher probability of dental care utilization than do males, a statistically significant 2.5 percent. And, females had 0.24 more visits than did males. Moreover, females had to pay out-of-pocket statistically significantly more than did males, by 193 baht. In terms of dental care treatments, females tended to use scaling, filling, dentures, and orthodontic services more than did males, statistically significant at 1.8, 2.7, 1.2, and 3.6 percent, respectively. In contrast, females tended to undergo extraction treatments less than males did, statistically significant at 7.5 percent.

As for comparisons of other age groups, age seems to be an important factor in determining which type of dental care treatment is provided. Just as with the comparison of those 65 years old and older, younger age groups, such as those 0–15 to 45–54 years, tend to undergo more scaling, filling, and orthodontic treatment. In contrast, younger age groups tend to have fewer extractions. The use of dental treatments may illustrate the nature of teeth conditions and age. For example, younger people tend to have fewer permanent teeth loss, which leads to them undergoing more treatments to maintain permanent teeth.

Regarding nationality, Thais have a higher probability of dental care utilization in the previous 12 months than do other nationalities and stateless persons, statistically significant at 4 percent. However, people of other nationalities or foreigners have to pay more out of pocket than do Thais, statistically significant at 664 baht.

As for the level of education, this factor seems to be particularly important. In general, people with a higher education had a higher probability of seeking dental care, visited the dentist more, and spent more in the previous 12 months than did those with less education. For example, compared with persons with no formal schooling, people who had completed high school and beyond had a higher probability of dental care utilization and more dental care visits, statistically significant at 2.4–7.1 percent and 0.3–0.4 visits, respectively. People with at least a bachelor's degree paid more out-of-pocket for dental care, statistically significant at 296–335 baht. Moreover, people with more education tended to employ preventive dental care and undergo restorative dental care, which helps save their permanent teeth, more than do uneducated persons. For instance, people who have completed a bachelor's or higher degree have a higher probability of undergoing oral exams, scalings, fillings, and root canals than do uneducated, statistically significant at 2.5–3.1, 17.8–18.1, 10.8–12.4, and 3.0–4.5 percent, respectively. Conversely, uneducated persons have a higher probability of extraction and needing denture services than those with more education, statistically significant at 8.3–30.7 and 2.9–6.5 percent, respectively. These estimations may indicate that Thai people who have undergone higher education seem to have received better oral health care knowledge and that they tend to have better oral hygiene and to take better care of their teeth, including going regularly to the dentist. That behavior helps prevent severe oral or dental disease, which in turn results in a lower probability of tooth loss or the need for extraction services.

With regard to marital status, both married and widowed/divorced/separated respondents had fewer dental visits than did those who were single, statistically significant at 0.15–0.23 visits. Both marital status groups compared with the single group had lower probabilities of undergoing scaling treatment, statistically significant 3.7–3.8 percent, while they had a higher probability of extraction, statistically significant 5.6–6.6 percent.

There were some occupations, for instance, unskilled workers, factory workers, crafts workers, and the unemployed, who had a lower probability of dental care utilization and scaling treatment (preventive care) than did public servants, statistically significant 1.4–1.9 percent and 5.3–12.2 percent, respectively. In contrast, sales/service workers, farmers, crafts persons, and unskilled laborers had a higher probability of extraction (restorative care) than did public servants, statistically significant 4.5–11.5 percent.

In terms of average monthly income per head, having a higher income seemed to have a positive effect on both dental care utilization and dental care expenditure. People with 1,000 baht additional monthly income had a higher probability of dental care utilization in the previous 12 months, statistically significant at 1 percent, and they tended to spend more out-of-pocket for dental services by 4 baht. Furthermore, an increase in monthly income had a positive effect on preventive care, such as oral exams and scaling treatment, and on orthodontics, but it had a negative effect on restorative care such as extractions.

Respondents who lived in the capital, Bangkok, had a higher probability of dental care utilization and spending out-of-pocket than did those who lived in other regions of Thailand, statistically significant at 1.2–1.6 percent and 165–395 baht, respectively. Meanwhile, people who lived in any of the four regions outside Bangkok tended to undergo relatively less preventive care yet tended to experience relatively more restorative care. For example, respondents living in three of the four regions (all but the Northeast) had a lower probability of undergoing oral exams and scaling compared to those living in Bangkok, statistically significant at 2.2–2.8 percent and 6.2–8.8 percent, respectively. In contrast, respondents in all four regions had a higher probability of extraction and denture services, statistically significant at 6.6–11.7 percent and 1.4–2.3 percent, respectively. However, respondents in the central, northern, and northeast regions tended to undergo orthodontic treatments more than did those who lived in Bangkok, statistically significant 1.4–3.9 percent.

The place of dental care utilization also affects dental care expenditure. People who went to a dentist at a university hospital, private hospital, private clinic, or at locally unqualified providers had to pay more out-of-pocket than did those who went to a dentist at a community hospital (a public hospital), by 1,128 baht, 868 baht, 754 baht, and 913 baht, respectively. Interestingly, people who received dental care at a mobile dental unit or school had a greater probability of getting preventive care, such as oral exams and scaling, than they did at community hospitals, statistically significant at 23–77.7 percent and 33.2 percent (except for care at schools), respectively. Many public and private organizations in Thailand offer basic dental care programs via schools and mobile dental units. Schools also seem to be a crucial place to impart oral health knowledge to Thai children [31]. Youtube as social media today provides information for children such as information about mouth sores [32]. When it comes to dentures, people had a higher probability of getting them from locally unqualified providers than at community hospitals, statistically significant 46.6 percent. People tended to go to a dentist for orthodontic services at university hospitals, private hospitals, and private clinics rather than at community hospitals, statistically significant 4.4–5.6 percent.

Even more important, dental insurance coverage is a crucial factor in determining a demand for dental care, when controlled for individuals' socioeconomic profile. Compared with SSS beneficiaries, those covered under UCS and CSMBS had a lower probability of dental care utilization in the previous 12 months, statistically significant at 3.6 percent and 1 percent, respectively. However, those covered by private dental insurance and by dental insurance paid by their employers were more likely to avail themselves of dental care, statistically significant at 3 and 14.8 percent, respectively. Conversely, UCS and CSMBS subscribers and those who paid by cash had more dental visits than those under SSS, statistically significant at 0.17, 0.23, and 0.99 visits, respectively. Not surprisingly, in terms of the number of dental visits, people enrolled in UCS and CSMBS tended to go to the dentist more often than did SSS subscribers because SSS provides relatively lower comprehensive benefits for associated costs and treatments, as indicated in Table 1.

Dental insurance coverage also affected individual dental care expenditure. People who were uninsured, insured but have to pay extra, and those covered by private insurance had to pay out-of-pocket for dental care more than did those enrolled in SSS, statistically significant at 2,104 baht, 2,107 baht, and 486 baht, respectively. On the other hand, people with generous coverage of comprehensive dental services seemed to save on out-of-pocket expenses. CSMBS subscribers, those covered by insurance paid by their employer, and those covered by other types of insurance had to pay less out-of-pocket than did those enrolled in SSS, statistically significant at 1,223 baht, 529 baht, and 1,621 baht, respectively.

As for preventive dental treatments, those covered by UCS and private dental insurance were more likely to undergo oral exams than were those under SSS, statistically significant at 2.1 and 9.9 percent, respectively. Interestingly, UCS and subscribers CSMBS, those covered by private insurance, and those who were insured but have to pay extra were less likely to get scaling treatments than were those under SSS, statistically significant at 11.1 percent, 3.4 percent, 18.0 percent, and 16.8 percent, respectively. It is possible that SSS covered the cost of essential dental services, including scaling, by fee-for-service that was no more than 900 baht in total per year per person. In this case, SSS subscribers might have chosen one service, such as scaling, even without any symptom of oral illness, before the end of the fiscal year.

In the case of basic restorative dental treatments, beneficiaries under UCS and those who were insured but paid in cash had a higher probability of extraction treatment than did those under SSS, with statistically significant 5.4 and 4.8 percent, respectively. This may be because even though SSS and UCS provide full coverage for extraction, UCS subscribers might tend to be informal or low-income workers with poor oral health. When visiting a dentist, their oral diseases might have already progressed to the worst stage, requiring tooth extraction [25]. Another possibility is that both those covered by UCS and those who were insured but have to pay extra had serious tooth disease already and thus tended to require more definitive dental treatment such as extractions instead of treatments that require a series of appointments and repeated visits to the dentist [24].

In terms of major restorative dental treatments, beneficiaries under CSMBS or dental private insurance and those who were insured but paid in cash were more likely to undergo of root canal treatment than were those covered by SSS, statistically significant at 1.3, 4.4, and 4.3 percent, respectively. This is because CSMBS provides generous coverage for root canal treatment, but SSS does not. Those who were insured but have to pay extra were more likely to be fitted for dentures than were those enrolled in SSS, statistically significant at 5.5 percent. The greater probability for those insured but paying in cash to undergo root canal and denture treatments may reflect inadequate coverage with UCS or CSMBS, thus requiring clients covered by these plans to pay out-of-pocket if they prefer more convenient services (without the long lines, inconvenient business hours, or other constraints) [30]. Also of interest, beneficiaries under UCS, CSMBS, private insurance, and those insured but have to pay extra had a higher probability of receiving orthodontic care than did those under SSS, statistically significant at 1.2, 1.6.7.5, and 9.3 percent, respectively. Although members of UCS and CSMBS tended to undergo orthodontics treatment more than did those under SSS, these services were apparently deemed necessary because both UCS and CSMBS provide coverage for cleft lip and palate patients, and it is SSS that does not. Both private insurance clients and those insured but have to pay extra made use of orthodontics for aesthetic/cosmetic purposes [33].

## 5. Conclusions and Policy Recommendations

This study investigates whether moral hazard in dental insurance exists as well as the impact of different types of dental insurance on the demand for dental care in Thailand, as a case study representative of other developing countries. Nationally representative data from Thailand Health and Welfare Surveys were used, covering 269,206 Thai people of all age groups. Taken into account also was the extent to which socioeconomic characteristics of individuals such as gender, age, level of education, marital status, level of income, and residential area affected demand for dental treatment.

Even though the estimation results did not find evidence of moral hazard in terms of the probability of dental care utilization and dental care expenditure in the 12 months previous to the surveys, we did find a moral hazard in the number of dental care visits and type of dental services.

People with generous insurance coverage, such as provided by UCS and CSMBS, had more dental visits than did those covered by SSS, statistically significant at 0.17–0.23 visits. In other words, people with greater coverage of dental benefits went to a dentist more often than those with lower coverage.

Dental insurance coverage also determined which type of dental care treatment was provided. People with generous insurance coverage, such as UCS and private insurance, tended to go for oral exams, which are preventive care, more than did those with relatively lower coverage, such as SSS, statistically significant at 2.1 and 9.9 percent, respectively. Regarding basic and major restorative treatments, UCS members tended to get extractions more than SSS members, statistically significant at 5.4 percent. People with CSMBS and private insurance also tended to undergo root canal treatments more than did those under SSS, statistically significant at 1.3 and 4.4 percent, respectively. This is consistent with the study results of Meyerhoefer et al. [9]; who found that having dental insurance increases the probability of using the three types of dental treatments (preventive, basic restorative, and major restorative care). This is due to the fact that the higher dental insurance coverage improves access to dental care and lowers out-of-pocket expenses, especially when getting needed and costly restorative services (root canals and dentures).

More interestingly, dental insurance is found to increase the use of preventive care (examination) in Thailand as a case of developing countries. The implication is that the essential and comprehensive dental care services that are provided full coverage (free plan) through the three main public health insurance schemes (UCS, SSS, and CSMBS) should be guaranteed benefits for everyone in order to promote regular access to dental care, especially preventive care that can prevent the severe oral disease from developing and therefore reduce the number and cost of treatments for more serious conditions, thus improving overall the population's well-being by helping people maintain good oral health.

However, there are still inequalities in dental benefits coverage among the three main public health insurance schemes. As seen in the findings of this study, people with generous dental coverage (UCS and CSMBS) tend to visit a dentist more often and avail themselves of essential dental treatments such as oral exams as well as other necessary but costly procedures, such as root canals, more than do those people with lower coverage (SSS). Beneficiaries under SSS, therefore, tend to face financial barriers that can limit access to dental care and can result in lower dental care utilization overall, a crucial aspect of healthcare utilization inequity in Thailand. Merging health insurance funds would be a worthwhile starting point for improving equity in healthcare financing and access to healthcare services, a solution suggested by Bazyar et al. [34]. Although this is not a new policy recommendation, it should nevertheless be put into practice even though doing so may not be easy.

In addition to socioeconomic, financial factors, and types of insurance addressed here, there are other issues, such as oral health status, self-perceived need for dental care, waiting time periods, travel costs, and paid sick leave, which might influence the demand for dental care. Unfortunately, these other factors are not included in this current study because of the limitations of the data sets. Further research could investigate these active factors to better understand the demand for dental care in a developing country context, especially in a country such as Thailand, where informal workers dominate half the labor market. More important, improved oral health, as one key outcome, may indicate whether additional dental care utilization is as efficient and far reaching as it could be when comparing before and after scenarios after more generous and expansive dental insurance coverage has been implemented. The study of the relationship between oral health outcomes and dental insurance coverage is thus a compelling topic for continued investigation.

Nowadays the demand for dental services in the pre-, during-, and post COVID-19 pandemic should also be given attention and further study. Although this study used the latest version of the data set (at the investigation moment) from the 2019 Health and Welfare Surveys in Thailand, the data had been collected before the spread of the disease. The lack of data sets about this issue is still a challenge.

## Figures and Tables

**Table 1:** Differences of dental benefits coverage among 3 health insurance schemes.

Dental services	CSMBS^*∗*^	UCS^*∗∗*^	SSS^*∗∗∗*^
Oral examination	Yes	Yes	Yes
Fluoride treatment	170 baht/visit for persons with a high risk of dental caries	Capitation budget for children and persons with a high risk of dental caries^*∗∗∗∗*^	No
Sealant treatment	No	Capitation budget for children and adolescents^*∗∗∗∗*^	No
Scaling	140 baht/visit for half of the mouth or 280 baht/visit for full mouth	Benefits of these basic dental services: Scaling, filling, extraction, and wisdom tooth removal are included in the outpatient (OP) capitation budget	Benefits cover the fee for service (FFS) of these basic dental services: Scaling, filling, extraction, and wisdom tooth removal but no more than 900 baht/person/year in total.
Filling	240–600 baht/tooth, depending on materials and number of cavities		
Extraction	200–350 baht/tooth, depending on complexity		
Wisdom tooth removal	700–1,000 baht/tooth		
Root canal treatment on primary teeth	500–970 baht/tooth, depending on teeth position	Benefit is included in the OP capitation budget	No
Root canal treatment on permanent teeth	1,060–3,500 baht/tooth, depending on teeth position	No	No
Removable partial dentures for 1–5 teeth	1,500 baht/person	Acrylic partial or complete dentures within capitation budget	1,300 baht/person
Removable partial dentures for 6 teeth +	2,000 baht/person		1,500 baht/person
Removable complete dentures for upper or lower mouth	3,000 baht/person		2,400 baht/person
Removable complete dentures for both upper and lower mouth	6,000 baht/person		4,400 baht/person
Orthodontics	Only cleft lip and palate patients and patients who have had accidents are allowed by a dentist to get benefits	48,000 baht/person over lifespan only for cleft lip and palate patients	No
Place of dental visits	Beneficiaries are able to visit a dentist at any public hospital.	Most beneficiaries are able to visit a dentist at public hospitals according to places in their contract.	Beneficiaries are able to visit a dentist at any private or public clinic and hospital according to their contract.

Source: ^*∗*^Comptroller General's Department [10] ^*∗∗*^ NHSO 2017, 2020 ^*∗∗∗*^Social Security Office [11] ^*∗∗∗∗*^Services are included in the capitation budget under UCS for all targeted Thai citizens regardless of health insurance schemes.

**Table 2:** Dental care utilization in the previous 12 Months with socioeconomic characteristics and dental insurance by tabulation.

Variables	The Number of Dental Care Visits					Dental Care Expenditure Deciles									
	0	1	2	3+	Observations		(0 baht)	(10–30)	(35–400)	(420–700)	(750–1,400)	(1,500+)	Average	Observation	
	Percent	Percent	Percent	Percent			Percent	Percent	Percent	Percent	Percent	Percent	Unit: baht		
Gender															
Male	93.5	5	1	0.4	127,949	(47.5%)	56.8	7.1	8.5	10.4	8.6	8.6	471.6	7,434	(38.4%)
Female	91	6.7	1.5	0.9	141,257	(52.5%)	53.5	8.4	7.6	9.2	10.6	10.6	573.7	11,932	(61.6%)
Total	92.2	5.9	1.3	0.7	269,206	(100%)	54.8	7.9	8	9.7	9.8	9.8	534.5	19,366	(100%)

Age															
0–15 years	89.3	8.4	1.7	0.6	51,707	(19.2%)	70.7	5	7.7	6.5	5.4	4.6	266.2	4,093	(21.1%)
16–24 years	91.7	5.4	1.3	1.6	25,386	(9.4%)	33.5	11.1	8.2	12	19.1	16	810	2,063	(10.7%)
25–34 years	92.3	5.5	1.2	1	29,857	(11.1%)	43.2	7.4	9	13.1	15.2	12.2	638.7	2,283	(11.8%)
35–44 years	93.2	5.3	1.1	0.4	39,186	(14.6%)	49.8	8.8	9.2	12.6	11	8.6	476.3	2,652	(13.7%)
45–54 years	92.9	5.5	1.1	0.5	46,284	(17.2%)	50	12.2	8.6	11.2	9.5	8.5	497.3	3,252	(16.8%)
55–64 years	92.4	5.8	1.3	0.5	39,847	(14.8%)	57.8	8.1	7.1	8.3	7.8	10.8	603.2	3,003	(15.5%)
65+ years	94.4	4.2	0.9	0.5	36,939	(13.7%)	66.9	2.7	5.7	5.5	5.4	13.8	713	2,020	(10.4%)
Total	92.2	5.9	1.3	0.7	269,206	(100%)	54.8	7.9	8	9.7	9.8	9.8	534.5	19,366	(100%)

Level of education															
No formal education	95	4.2	0.5	0.2	12,448	(4.9%)	68.4	8.8	7.4	5.5	4.7	5.3	295.4	512	(2.8%)
Preschool	90.6	7.6	1.4	0.5	54,106	(21.5%)	71.2	7.1	7.8	5.4	3.6	4.9	272.2	4,056	(21.8%)
Primary school	94.5	4.3	0.8	0.4	86,636	(34.4%)	57.5	12.7	7.5	8.3	6.4	7.6	445	4,514	(24.3%)
Secondary school	93.1	5	1	0.8	34,581	(13.7%)	43.2	11.5	8.6	11.6	13.6	11.4	648	2,332	(12.5%)
High school	91.5	6.1	1.3	1	30,843	(12.3%)	41.3	9.3	10	12.4	14.1	13	675.2	2,583	(13.9%)
Diploma	89.4	7.8	1.8	1	8,237	(3.3%)	48	4.6	8.5	13.6	12.9	12.5	648.7	863	(4.6%)
Bachelor's degree	85.7	9.7	2.9	1.7	21,831	(8.7%)	44.9	1.8	7.4	13.6	16.6	15.6	821.1	3,117	(16.7%)
Higher than bachelor's degree	79.3	12.8	5.7	2.2	3,074	(1.2%)	48.7	0.3	4.4	11.7	17.4	17.4	870.3	632	(3.4%)
Total	92	6	1.3	0.7	251,756	(100%)	53.9	8.1	8	9.8	10.1	10.1	547.5	18,609	(100%)

Marital status															
Single	91.4	5.8	1.5	1.3	54,457	(23.9%)	40.7	7	7.5	12.7	17.4	14.7	764.7	4,506	(28%)
Married	93	5.3	1.1	0.5	141,456	(62%)	53.6	9.7	8.1	10.1	8.9	9.6	534.4	9,724	(60.5%)
Widowed/Divorced/Separated	94.2	4.5	0.9	0.5	32,059	(14.1%)	59.9	7.4	7.9	7.3	6.8	10.6	583.9	1,839	(11.4%)
Total	92.8	5.3	1.2	0.7	227,972	(100%)	50.7	8.7	7.9	10.5	11.1	11.2	604.6	16,069	(100%)

Occupation															
Public servants	86.6	9.2	3	1.2	4,173	(1.9%)	41.3	2.9	6.5	13.9	16.7	18.7	877.8	552	(3.6%)
Professions	84	10.6	3.6	1.8	7,657	(3.5%)	55.5	0.6	6.1	11.8	14.3	11.7	643.8	1,223	(7.9%)
Technicians	86.9	9	2.8	1.4	4,701	(2.1%)	50.7	0.3	8.3	14	13.8	12.8	731.1	615	(4%)
Clerks	87.3	9	2.1	1.6	4,870	(2.2%)	47.1	1.5	11.6	13	13.8	13	689	614	(4%)
Service/Sales workers	91.5	6.3	1.4	0.8	30,832	(14%)	39.7	6.9	10	13.8	14.7	14.9	790.9	2,595	(16.7%)
Farmers	94.7	4.3	0.7	0.3	59,136	(26.8%)	58.7	17.8	7.5	6.5	4.2	5.4	315.8	3,057	(19.7%)
Crafts	94.5	4.3	0.9	0.4	14,681	(6.7%)	53.7	9.4	8.6	12.4	8.3	7.7	471.2	807	(5.2%)
Factory workers	94.1	4.8	0.9	0.3	10,214	(4.6%)	54.8	5.3	11.4	13.4	9.5	5.7	371.6	599	(3.9%)
Unskilled workers	95.5	3.6	0.6	0.3	16,565	(7.5%)	56.8	16	9.2	8.1	5.6	4.4	311.9	732	(4.7%)
Unemployed	92.9	5	1.2	0.9	67,656	(30.7%)	48.8	7.8	6.8	9.5	13	14.1	727.7	4,709	(30.4%)
Total	92.9	5.3	1.2	0.7	220,485	(100%)	50.4	8.7	8	10.5	11.1	11.3	607.6	15,503	(100%)

Average monthly income quintiles (unit: Baht)															
Q1 (773 baht)	94.5	4.1	0.8	0.6	34,688	(21.6%)	54.1	11.8	7.8	7.9	8.8	9.6	530.4	1,858	(16.4%)
Q2 (3,956 baht)	94	4.5	0.9	0.6	33,465	(20.8%)	55.5	11.4	8.5	8.1	7.6	8.9	507.8	1,976	(17.4%)
Q3 (7,426 baht)	94.4	4.3	0.8	0.5	30,710	(19.1%)	52	12.6	11.3	9.2	7.7	7.3	421.3	1,693	(14.9%)
Q4 (12,336 baht)	92.5	5.7	1.1	0.6	30,151	(18.8%)	44.8	7.1	11.3	14.6	11.3	10.9	626.4	2,225	(19.6%)
Q5 (32,375 baht)	88.5	8	2.4	1.1	31,588	(19.7%)	47.9	2.8	6.8	13	14	15.4	804.1	3,606	(31.7%)
Total	92.8	5.3	1.2	0.7	160,602	(100%)	50.3	8.1	8.8	11	10.6	11.3	615.9	11,358	(100%)

Dental insurance															
Uninsured	95.3	3.2	1	0.5	1,990	(0.8%)	5.1	1.3	11.5	32.1	17.9	32.1	1,646.20	78	(0.4%)
UCS	94.9	4.2	0.7	0.2	197,020	(76.4%)	79.5	17.5	1.7	0.5	0.3	0.5	29.5	8,595	(44.4%)
SSS	91.8	6.7	1.3	0.2	25,787	(10%)	67.1	0.5	11.7	10.5	6.8	3.4	252	2,116	(10.9%)
CSMBS	90.7	6.7	2	0.7	24,366	(9.4%)	92.9	0.4	2.4	1	1.4	1.8	96.5	2,219	(11.5%)
Private insurance	86.1	10.4	2	1.5	941	(0.4%)	59.5	0	3.1	14.5	6.9	16	717.8	131	(0.7%)
Insurance by employer	86.5	8.9	3.7	0.9	694	(0.3%)	77.7	0	2.1	8.5	3.2	8.5	305.9	94	(0.5%)
Other insurance	92	6.2	1.3	0.4	1,043	(0.4%)	89.3	6.7	2.7	0	0	1.3	36	75	(0.4%)
Insured paid by cash	0	63.7	18.9	17.4	6,089	(2.4%)	1.1	0.1	17.8	25.3	27.7	27.9	1,501.50	6,034	(31.2%)
Total	91.9	6.1	1.3	0.7	257,930	(100%)	54.8	7.9	8	9.7	9.8	9.8	533.3	19,342	(100%)

Region															
Bangkok	85.3	9.5	3.7	1.5	12,152	(4.5%)	30.9	0.4	5.7	15.8	22.1	25.2	1,197.60	1,713	(8.8%)
Central	92.6	5.4	1.3	0.7	78,624	(29.2%)	50.8	6.2	8.7	11.5	11.4	11.4	604.8	5,512	(28.5%)
Northern	91.4	6.7	1.1	0.7	57,875	(21.5%)	62.4	8.8	7.6	6.8	7	7.4	403.6	4,461	(23%)
Northeast	93.5	5.3	0.8	0.4	73,755	(27.4%)	66.5	10.6	6.9	6.1	5.1	4.8	298.1	4,279	(22.1%)
Southern	92.1	5.7	1.5	0.7	46,800	(17.4%)	48.5	10	9.9	11.9	10.7	9	555.5	3,401	(17.6%)
Total	92.2	5.9	1.3	0.7	269,206	(100%)	54.8	7.9	8	9.7	9.8	9.8	534.5	19,366	(100%)

**Table 3:** Use of dental treatments in the previous 12 Months with socioeconomic characteristics and dental insurance by tabulation.

Variables	Preventive Treatments				Basic Restorative		Major Restorative			Observation	
	Exams	Scaling	Sealant	Fluoride	Filling	Extraction	Root canal	Denture	Orthodontics		
	Percent	Percent	Percent	Percent	Percent	Percent	Percent	Percent	Percent		
Gender											
Male	46.1	35.8	40	48.8	35.7	42.8	42.2	35.7	20.7	8,233	(39.3%)
Female	53.9	64.2	60	51.2	64.3	57.2	57.8	64.3	79.3	12,711	(60.7%)
Total	100	100	100	100	100	100	100	100	100	20,944	(100%)

Age											
0–15 years	64.5	12.6	41.9	97.6	28.3	19.6	16.3	0.1	10.2	5,519	(26.4%)
16–24 years	4.1	13.4	8.6	0.5	12.5	5.4	9.8	1.7	50.1	2,102	(10%)
25–34 years	4.7	18.5	9.5	0.2	12.7	6.6	6.6	1	30	2,283	(10.9%)
35–44 years	4.2	21.4	9.5	0.5	15	10.6	16.3	4.8	7.9	2,671	(12.8%)
45–54 years	5.9	18.4	14.3	0.3	15.6	20.5	19.1	13.6	1.5	3,278	(15.7%)
55–64 years	8.6	11.4	9.5	0.8	11.3	21.7	22.3	31.3	0	3,043	(14.5%)
65+ years	7.9	4.2	6.7	0.2	4.6	15.6	9.6	47.5	0.2	2,048	(9.8%)
Total	100	100	100	100	100	100	100	100	100	20,944	(100%)

Level of education											
No formal education	6.5	1.2	4	12	1.5	4	2	5.5	0.2	625	(3.1%)
Nursery school	51.5	9.7	31.7	76.3	22.3	31.8	11	27.6	0.1	5,088	(25.4%)
Primary school	19.1	15.8	19.8	8.3	21.6	33.8	18.8	35.7	8.4	4,715	(23.6%)
Secondary school	5.2	13	10.9	0.9	14.2	11.1	12.2	8.8	25.9	2,372	(11.9%)
High school	5.6	17.7	9.9	0	15.5	9.5	16	8.4	27.7	2,588	(12.9%)
Diploma	2	6.7	4	0.9	4.9	2.9	5.6	3	7	866	(4.3%)
Bachelor's degree	8.1	29.3	16.8	1.4	16.7	6.2	25.2	9.5	26.6	3,117	(15.6%)
Higher than bachelor's degree	2.2	6.5	3	0.3	3.3	0.7	9.2	1.4	4	633	(3.2%)
Total	100	100	100	100	100	100	100	100	100	20,004	(100%)

Marital status											
Single	29.4	36.8	44.4	66.7	35.5	13.9	27	6.9	74.2	4,678	(28.6%)
Married	57.6	55.5	46	30.3	56	70.3	64.7	68.3	23	9,788	(59.9%)
Widowed/Divorced/Separated	13	7.7	9.5	3	8.5	15.8	8.3	24.8	2.8	1,874	(11.5%)
Total	100	100	100	100	100	100	100	100	100	16,340	(100%)

Occupation											
Public servants	3.5	6	3.2	6.7	3.5	1.8	5.9	2.2	1.8	555	(3.5%)
Professionals	7.9	14.4	8.1	20	7.7	2.2	14.8	1.8	10.1	1,224	(7.8%)
Technicians	2.9	7.2	6.5	0	4.1	1.4	3.3	1.7	5.7	615	(3.9%)
Clerks	1.7	6.8	4.8	6.7	4.7	1.6	2.8	0.8	6.5	617	(3.9%)
Service/Sales workers	11.6	17.2	29	20	19.4	15.6	20.9	15.3	14.9	2,607	(16.6%)
Farmers	21.4	10.3	6.5	13.3	16.1	32.6	11	21	3.2	3,119	(19.9%)
Crafts	3.4	4.2	1.6	0	5	6.6	3.5	6.6	2.9	807	(5.2%)
Factory workers	1.7	4.7	9.7	0	4.7	3.6	2.1	1.8	2.6	602	(3.8%)
Unskilled workers	3.6	3.6	3.2	0	4.1	6.9	1.9	4.8	1	739	(4.7%)
Unemployed	42.3	25.5	27.4	33.3	30.4	27.8	33.8	44	51.2	4,783	(30.5%)
Total	100	100	100	100	100	100	100	100	100	15,668	(100%)

Average monthly income quintiles (unit: Baht)											
Q1 (773 baht)	22.7	11.1	12.5	18.2	15.5	18.9	13.4	22.1	24.6	1,895	(16.5%)
Q2 (3,956 baht)	19.3	10.6	16.7	9.1	15.1	23.3	9.8	24.1	20.9	2,018	(17.6%)
Q3 (7,426 baht)	14.8	11.5	12.5	9.1	13.7	19.1	10.7	13.9	12	1,718	(15%)
Q4 (12,336 baht)	14.4	20.4	31.3	9.1	22.6	19.1	13.4	14.9	19.3	2,235	(19.5%)
Q5 (32,375 baht)	28.7	46.4	27.1	54.5	33	19.6	52.7	25	23.3	3,609	(31.5%)
Total	100	100	100	100	100	100	100	100	100	11,475	(100%)

Dental insurance											
Uninsured	0.9	0.5	1	0.3	0.4	0.2	0.8	0.3	0.2	92	(0.4%)
UCS	73	29.4	37.1	83	45.6	59.9	21.6	34.1	1.6	9,971	(48%)
SSS	2.6	22.5	4.8	0.3	12.1	6	4.6	2.2	0.4	2,110	(10.1%)
CSMBS	10.7	16	10.5	4.7	11.5	7.9	18	13.1	0.9	2,272	(10.9%)
Private insurance	0.8	0.7	1	1.2	0.7	0.4	1.6	0.4	0.5	130	(0.6%)
Insurance by employer	0.2	1.2	1.9	0.2	0.4	0	0.6	0.3	0.2	94	(0.5%)
Other insurance	0.5	0.3	0	0.2	0.5	0.5	0	0.1	0.4	83	(0.4%)
Insured paid by cash	11.2	29.5	43.8	10.2	28.8	25.1	52.9	49.5	95.8	6,041	(29.1%)
Total	100	100	100	100	100	100	100	100	100	20,793	(100%)

Region											
Bangkok	7.1	15.5	10.5	3	8.5	3.5	15.3	6.2	11.1	1,768	(8.4%)
Central	18	31.1	27.6	25.4	30.9	26	28.5	31.4	31.5	5,771	(27.6%)
Northern	29.3	20.4	23.8	15.5	22.9	24.6	18.7	28.2	24.4	4,935	(23.6%)
Northeast	30.6	16.9	14.3	30.1	18.2	27.8	18.7	17.1	20.2	4,800	(22.9%)
Southern	15	16.2	23.8	25.9	19.6	18.1	18.7	17.1	12.8	3,670	(17.5%)
Total	100	100	100	100	100	100	100	100	100	20,944	(100%)

Authors' Calculation. Source: Thailand's National Statistical Office.

**Table 4:** Estimated results of dental care utilization, expenditure, and use of dental treatments in the previous 12 Months.

Variables	Dental Care	^#^ Dental	Dental Care	Preventive				Basic Restorative		Major Restorative		
	Utilization	Visits	Expenditure	Exam	Scaling	Sealant	Fluoride	Filling	Extraction	Root canal	Denture	Orthodontic
	(1)	(2)	(3)	(4)	(5)	(6)	(7)	(8)	(9)	(10)	(11)	(12)
	Probit	Tobit	Tobit	Probit	Probit	Probit	Probit	Probit	Probit	Probit	Probit	Probit
Gender (reference: Male)												

Female	0.025^*∗∗∗*^	0.244^*∗∗∗*^	193.115^*∗∗∗*^	0.005	0.018^*∗∗*^	−0.003^*∗*^	0.002	0.027^*∗∗∗*^	−0.075^*∗∗∗*^	−0.004	0.012^*∗∗∗*^	0.036^*∗∗∗*^
	*(0.001)*	*(0.048)*	*(46.284)*	*(0.005)*	*(0.009)*	*(0.002)*	*(0.002)*	*(0.008)*	*(0.009)*	*(0.004)*	*(0.004)*	*(0.005)*

Age (reference: 65 years and over)												
0–15 years	0.009	0.711^*∗∗∗*^	−474.685^*∗∗*^	−0.017	0.201^*∗∗∗*^	−0.001	—	0.280^*∗∗∗*^	−0.304^*∗∗∗*^	−0.011	—	0.113^*∗∗∗*^
	*(0.007)*	*(0.221)*	*(207.016)*	*(0.022)*	*(0.042)*	*(0.005)*	—	*(0.046)*	*(0.031)*	*(0.013)*	—	*(0.026)*

16–24 years	−0.010^*∗∗∗*^	0.748^*∗∗∗*^	−345.222^*∗∗∗*^	−0.015	0.155^*∗∗∗*^	−0.002	0.005	0.109^*∗∗∗*^	−0.174^*∗∗∗*^	−0.014^*∗*^	−0.157^*∗∗∗*^	0.125^*∗∗∗*^
	*(0.003)*	*(0.114)*	*(107.879)*	*(0.012)*	*(0.019)*	*(0.003)*	*(0.007)*	*(0.018)*	*(0.021)*	*(0.007)*	*(0.012)*	*(0.012)*

25–34 years	−0.016^*∗∗∗*^	0.446^*∗∗∗*^	−328.846^*∗∗∗*^	−0.004	0.168^*∗∗∗*^	−0.001	—	0.090^*∗∗∗*^	−0.134^*∗∗∗*^	−0.018^*∗∗∗*^	^*∗∗∗*^	0.093^*∗∗∗*^
	*(0.003)*	*(0.103)*	*(99.121)*	*(0.011)*	*(0.018)*	*(0.003)*	—	*(0.015)*	*(0.020)*	*(0.007)*	*(0.012)*	*(0.008)*

35–44 years	−0.012^*∗∗∗*^	−0.097	−438.216^*∗∗∗*^	−0.027^*∗∗∗*^	0.213^*∗∗∗*^	−0.001	−0.001	0.092^*∗∗∗*^	−0.116^*∗∗∗*^	−0.001	−0.146^*∗∗∗*^	0.027^*∗∗∗*^
	*(0.002)*	*(0.093)*	*(92.205)*	*(0.009)*	*(0.016)*	*(0.003)*	*(0.002)*	*(0.013)*	*(0.018)*	*(0.007)*	*(0.013)*	*(0.006)*

45–54 years	−0.004^*∗*^	−0.103	−190.108^*∗∗*^	−0.028^*∗∗∗*^	0.154^*∗∗∗*^	0.001	−0.000	0.083^*∗∗∗*^	−0.035^*∗∗*^	0.003	−0.136^*∗∗∗*^	0.003
	*(0.002)*	*(0.082)*	*(82.675)*	*(0.008)*	*(0.014)*	*(0.003)*	*(0.002)*	*(0.012)*	*(0.016)*	*(0.007)*	*(0.012)*	*(0.003)*

55–64 years	0.005^*∗∗*^	−0.132^*∗*^	−127.115	−0.011	0.074^*∗∗∗*^	0.000	0.002	0.053^*∗∗∗*^	0.002	0.012^*∗*^	−0.090^*∗∗∗*^	—
	*(0.002)*	*(0.077)*	*(78.895)*	*(0.007)*	*(0.013)*	*(0.003)*	*(0.003)*	*(0.011)*	*(0.015)*	*(0.007)*	*(0.012)*	—

Religion (reference: Buddhism)												
Islam	−0.002	−0.212^*∗*^	−40.886	−0.014	−0.027	0.001	—	0.055^*∗∗∗*^	0.007	0.002	0.006	−0.025^*∗∗*^
	*(0.003)*	*(0.114)*	*(106.007)*	*(0.010)*	*(0.020)*	*(0.003)*	—	*(0.020)*	*(0.021)*	*(0.009)*	*(0.012)*	*(0.011)*

Christianity	−0.006	−0.131	294.897	0.039	0.042	0.024	—	−0.027	−0.013	−0.011	—	−0.011
	*(0.006)*	*(0.290)*	*(287.311)*	*(0.033)*	*(0.054)*	*(0.026)*	—	*(0.045)*	*(0.054)*	*(0.018)*	—	*(0.031)*

Other religions	−0.012	−0.848	−943.448	—	0.334	—	—	0.084	—	—	—	—
	*(0.035)*	*(1.357)*	*(1,081.451)*	—	*(0.260)*	—	—	*(0.240)*	—	—	—	—

Nationality (reference: Thai)												
Other nationalities	−0.042^*∗∗∗*^	0.088	663.722^*∗*^	0.077	−0.148^*∗∗∗*^	—	—	0.009	0.115	0.048	−0.005	0.087
	*(0.002)*	*(0.403)*	*(360.105)*	*(0.064)*	*(0.055)*	—	—	*(0.068)*	*(0.075)*	*(0.058)*	*(0.040)*	*(0.067)*

Stateless	−0.041^*∗∗∗*^	0.116	−6,801.716	—	—	—	—	—	—	—	—	—
	*(0.009)*	*(2.299)*	*(229,756.698)*	—	—	—	—	—	—	—	—	—

Level of Education (Reference: No formal education)												
Preschool	0.013^*∗∗∗*^	0.181	−150.149	0.005	−0.017	—	—	0.008	0.025	−0.006	−0.027^*∗*^	−
	*(0.003)*	*(0.156)*	*(166.064)*	*(0.012)*	*(0.029)*	—	—	*(0.021)*	*(0.031)*	*(0.007)*	*(0.016)*	*−*

Primary school	0.009^*∗∗∗*^	0.222	118.194	0.002	0.007	—	—	0.045^*∗∗*^	0.004	0.010	−0.033^*∗∗*^	−0.006
	*(0.002)*	*(0.152)*	*(159.311)*	*(0.012)*	*(0.028)*	—	—	*(0.021)*	*(0.030)*	*(0.007)*	*(0.016)*	*(0.025)*

Secondary school	0.019^*∗∗∗*^	0.200	240.452	−0.001	0.081^*∗∗∗*^	—	—	0.075^*∗∗∗*^	−0.083^*∗∗*^	0.023^*∗∗∗*^	−0.029^*∗*^	0.025
	*(0.003)*	*(0.162)*	*(167.028)*	*(0.013)*	*(0.030)*	—	—	*(0.022)*	*(0.033)*	*(0.009)*	*(0.017)*	*(0.025)*

High school	0.024^*∗∗∗*^	0.297^*∗*^	243.296	−0.002	0.141^*∗∗∗*^	—	—	0.104^*∗∗∗*^	−0.162^*∗∗∗*^	0.028^*∗∗∗*^	−0.046^*∗∗∗*^	0.016
	*(0.003)*	*(0.161)*	*(165.983)*	*(0.013)*	*(0.030)*	—	—	*(0.022)*	*(0.032)*	*(0.009)*	*(0.017)*	*(0.025)*

Diploma	0.034^*∗∗∗*^	0.304^*∗*^	179.218	0.015	0.135^*∗∗∗*^	—	—	0.091^*∗∗∗*^	−0.161^*∗∗∗*^	0.033^*∗∗∗*^	−0.038^*∗∗*^	0.022
	*(0.004)*	*(0.179)*	*(182.100)*	*(0.016)*	*(0.034)*	—	—	*(0.026)*	*(0.036)*	*(0.011)*	*(0.019)*	*(0.026)*

Bachelor's degree	0.039^*∗∗∗*^	0.417^*∗∗*^	295.624^*∗*^	0.025^*∗*^	0.178^*∗∗∗*^	—	—	0.108^*∗∗∗*^	−0.241^*∗∗∗*^	0.030^*∗∗∗*^	−0.059^*∗∗∗*^	0.039
	*(0.004)*	*(0.165)*	*(169.245)*	*(0.014)*	*(0.031)*	—	—	*(0.023)*	*(0.033)*	*(0.009)*	*(0.016)*	*(0.025)*

Higher than bachelor's degree	0.071^*∗∗∗*^	0.444^*∗∗*^	334.758^*∗*^	0.031^*∗*^	0.181^*∗∗∗*^	—	—	0.124^*∗∗∗*^	−0.307^*∗∗∗*^	0.045^*∗∗∗*^	−0.065^*∗∗∗*^	0.040
	*(0.008)*	*(0.195)*	*(195.491)*	*(0.019)*	*(0.037)*	—	—	*(0.030)*	*(0.037)*	*(0.013)*	*(0.018)*	*(0.028)*

Marital status (reference: Single)												
Married	0.001	−0.150^*∗∗*^	−58.967	0.018^*∗∗∗*^	−0.037^*∗∗∗*^	−0.005^*∗*^	0.002	0.008	0.056^*∗∗∗*^	0.005	0.013^*∗*^	−0.019^*∗∗∗*^
	*(0.002)*	*(0.067)*	*(62.397)*	*(0.006)*	*(0.012)*	*(0.003)*	*(0.002)*	*(0.010)*	*(0.013)*	*(0.005)*	*(0.007)*	*(0.006)*

Widowed/Divorced/Separated	−0.007^*∗∗∗*^	−0.231^*∗∗∗*^	5.112	0.011	−0.038^*∗∗*^	−0.003	0.000	0.003	0.066^*∗∗∗*^	−0.000	0.011	0.005
	*(0.002)*	*(0.089)*	*(85.966)*	*(0.008)*	*(0.016)*	*(0.003)*	*(0.002)*	*(0.014)*	*(0.017)*	*(0.006)*	*(0.008)*	*(0.015)*

Occupation (reference: Public servant)												
Professional	−0.003	−0.081	−102.815	0.000	−0.008	0.001	0.005	−0.017	−0.012	0.010	−0.015	0.002
	*(0.005)*	*(0.136)*	*(128.249)*	*(0.013)*	*(0.025)*	*(0.003)*	*(0.009)*	*(0.019)*	*(0.029)*	*(0.008)*	*(0.014)*	*(0.015)*

Technician	−0.006	−0.009	−12.272	−0.002	−0.007	0.003	—	−0.004	−0.038	−0.003	0.014	0.003
	*(0.005)*	*(0.158)*	*(147.025)*	*(0.015)*	*(0.029)*	*(0.004)*	—	*(0.023)*	*(0.033)*	*(0.009)*	*(0.019)*	*(0.017)*

Clerk	−0.011^*∗∗*^	0.037	43.660	−0.026^*∗*^	−0.042	0.006	—	0.021	0.024	−0.002	−0.004	−0.014
	*(0.005)*	*(0.162)*	*(148.119)*	*(0.014)*	*(0.029)*	*(0.005)*	—	*(0.024)*	*(0.034)*	*(0.009)*	*(0.020)*	*(0.016)*

Sales/Service worker	−0.016^*∗∗∗*^	−0.196	18.739	−0.005	−0.062^*∗∗∗*^	0.006^*∗∗*^	−0.001	0.045^*∗∗*^	0.045^*∗*^	0.009	−0.008	−0.008
	*(0.004)*	*(0.126)*	*(115.603)*	*(0.012)*	*(0.023)*	*(0.003)*	*(0.006)*	*(0.019)*	*(0.025)*	*(0.007)*	*(0.012)*	*(0.014)*

Farmer	−0.007	−0.034	124.620	−0.007	−0.106^*∗∗∗*^	0.000	−0.003	0.018	0.095^*∗∗∗*^	0.001	−0.006	−0.008
	*(0.005)*	*(0.131)*	*(122.859)*	*(0.012)*	*(0.024)*	*(0.002)*	*(0.006)*	*(0.020)*	*(0.026)*	*(0.008)*	*(0.012)*	*(0.017)*

Crafts person	−0.014^*∗∗∗*^	−0.077	−47.868	−0.001	−0.117^*∗∗∗*^	−0.001	—	−0.001	0.112^*∗∗∗*^	−0.001	0.029^*∗*^	0.013
	*(0.005)*	*(0.149)*	*(139.763)*	*(0.014)*	*(0.027)*	*(0.002)*	—	*(0.022)*	*(0.029)*	*(0.009)*	*(0.016)*	*(0.020)*

Factory worker	−0.018^*∗∗∗*^	−0.105	−97.728	−0.024	−0.092^*∗∗∗*^	0.011∗	—	0.040	0.073^*∗∗*^	0.004	0.014	−0.001
	*(0.005)*	*(0.162)*	*(150.567)*	*(0.015)*	*(0.029)*	*(0.007)*	—	*(0.025)*	*(0.032)*	*(0.011)*	*(0.019)*	*(0.020)*

Unskilled worker	−0.019^*∗∗∗*^	0.098	130.442	−0.003	−0.122^*∗∗∗*^	0.001	—	0.005	0.115^*∗∗∗*^	0.004	0.028^*∗*^	−0.025
	*(0.005)*	*(0.157)*	*(149.083)*	*(0.015)*	*(0.028)*	*(0.003)*	—	*(0.024)*	*(0.031)*	*(0.011)*	*(0.017)*	*(0.023)*

Unemployed	−0.015^*∗∗∗*^	0.034	75.175	0.022^*∗*^	−0.053^*∗∗*^	0.002	−0.002	0.017	0.021	0.010	0.002	0.005
	*(0.005)*	*(0.129)*	*(118.801)*	*(0.012)*	*(0.024)*	*(0.002)*	*(0.006)*	*(0.019)*	*(0.025)*	*(0.007)*	*(0.012)*	*(0.015)*
Average Monthly Income (unit: baht)	0.001^*∗∗∗*^	0.001	0.004^*∗∗∗*^	0.001^*∗∗*^	0.001^*∗∗∗*^	0.001	−0.001	−0.001	−0.001^*∗∗∗*^	0.001	0.001	0.001^*∗∗*^
	*(0.000)*	*(0.000)*	*(0.001)*	*(0.000)*	*(0.000)*	*(0.000)*	*(0.000)*	*(0.000)*	*(0.000)*	*(0.000)*	*(0.000)*	*(0.000)*

Municipal area (reference: Outside municipal area)												
Within municipal area	−0.005^*∗∗∗*^	0.042	−16.866	−0.003	0.022^*∗∗*^	0.001	0.000	0.010	−0.030^*∗∗∗*^	−0.001	0.000	0.004
	*(0.001)*	*(0.049)*	*(47.606)*	*(0.005)*	*(0.009)*	*(0.001)*	*(0.002)*	*(0.008)*	*(0.009)*	*(0.004)*	*(0.004)*	*(0.006)*

Region (reference: Bangkok)												
Central	−0.016^*∗∗∗*^	0.126	−247.155^*∗∗∗*^	−0.026^*∗∗*^	−0.062^*∗∗∗*^	0.002	0.001	0.009	0.066^*∗∗∗*^	−0.005	0.019^*∗∗∗*^	0.014^*∗*^
	*(0.003)*	*(0.085)*	*(75.183)*	*(0.010)*	*(0.016)*	*(0.002)*	*(0.003)*	*(0.014)*	*(0.017)*	*(0.006)*	*(0.007)*	*(0.008)*

Northern	−0.005	0.309^*∗∗∗*^	−343.530^*∗∗∗*^	−0.028^*∗∗∗*^	−0.076^*∗∗∗*^	0.002	−0.001	−0.031^*∗∗*^	0.092^*∗∗∗*^	−0.003	0.023^*∗∗∗*^	0.039^*∗∗∗*^
	*(0.003)*	*(0.090)*	*(80.814)*	*(0.010)*	*(0.016)*	*(0.002)*	*(0.002)*	*(0.014)*	*(0.017)*	*(0.006)*	*(0.007)*	*(0.009)*

Northeast	−0.014^*∗∗∗*^	0.100	−394.861^*∗∗∗*^	−0.013	−0.084^*∗∗∗*^	−0.000	0.000	−0.054^*∗∗∗*^	0.117^*∗∗∗*^	−0.004	0.014∗	0.028^*∗∗∗*^
	*(0.003)*	*(0.093)*	*(84.790)*	*(0.011)*	*(0.017)*	*(0.002)*	*(0.003)*	*(0.015)*	*(0.018)*	*(0.007)*	*(0.007)*	*(0.009)*

Southern	−0.012^*∗∗∗*^	0.087	−164.938^*∗*^	−0.022^*∗∗*^	−0.088^*∗∗∗*^	0.003	0.002	−0.006	0.103^*∗∗∗*^	−0.002	0.022^*∗∗∗*^	0.006
	*(0.003)*	*(0.096)*	*(85.147)*	*(0.011)*	*(0.017)*	*(0.002)*	*(0.004)*	*(0.016)*	*(0.018)*	*(0.007)*	*(0.008)*	*(0.009)*

Year of dental care visits (reference: 2015)												
2017	0.026^*∗∗∗*^	0.131^*∗∗*^	−152.943^*∗∗*^	0.006	−0.025^*∗∗*^	−0.002	0.002	0.003	0.022^*∗∗*^	−0.010^*∗∗*^	−0.004	0.017^*∗*^
	*(0.002)*	*(0.061)*	*(59.915)*	*(0.006)*	*(0.011)*	*(0.002)*	*(0.003)*	*(0.010)*	*(0.011)*	*(0.004)*	*(0.005)*	*(0.009)*

2019	0.013^*∗∗∗*^	0.007	−215.100^*∗∗∗*^	0.006	−0.005	−0.003^*∗∗*^	−	−0.008	0.017	−0.008^*∗*^	−0.000	0.012
	*(0.002)*	*(0.065)*	*(64.219)*	*(0.006)*	*(0.012)*	*(0.001)*	*−*	*(0.010)*	*(0.012)*	*(0.004)*	*(0.006)*	*(0.010)*

Place of dental visits (reference: Community hospital)												
Health center	—	−0.044	−958.004^*∗∗∗*^	0.076^*∗∗∗*^	0.041^*∗∗∗*^	−0.007	0.003	−0.013	−0.054^*∗∗∗*^	−0.018^*∗∗*^	−0.037^*∗∗∗*^	—
	—	*(0.079)*	*(98.354)*	*(0.011)*	*(0.015)*	*(0.005)*	*(0.004)*	*(0.012)*	*(0.014)*	*(0.007)*	*(0.006)*	—

Public general/regional hospital	—	−0.076	−53.822	0.015^*∗∗*^	0.012	−0.000	0.001	0.027^*∗∗*^	−0.052^*∗∗∗*^	0.003	0.005	−0.004
	—	*(0.067)*	*(72.960)*	*(0.006)*	*(0.012)*	*(0.004)*	*(0.002)*	*(0.011)*	*(0.012)*	*(0.007)*	*(0.006)*	*(0.010)*

University hospital	—	0.376	1,127.807^*∗∗∗*^	−0.015	0.109^*∗∗*^	0.002	0.026	−0.033	−0.180^*∗∗∗*^	0.031	−0.018	0.056^*∗*^
	—	*(0.251)*	*(234.740)*	*(0.018)*	*(0.046)*	*(0.013)*	*(0.029)*	*(0.034)*	*(0.051)*	*(0.025)*	*(0.022)*	*(0.032)*

Private hospital	—	−0.160	868.169^*∗∗∗*^	0.023	0.014	−0.007	0.013	0.037	−0.104^*∗∗∗*^	−0.004	−0.015	0.044^*∗∗*^
	—	*(0.155)*	*(141.857)*	*(0.017)*	*(0.026)*	*(0.006)*	*(0.015)*	*(0.025)*	*(0.031)*	*(0.012)*	*(0.015)*	*(0.018)*

Private clinic	—	−0.107	754.169^*∗∗∗*^	−0.023^*∗∗∗*^	0.044^*∗∗*^	−0.007	0.000	0.017	−0.067^*∗∗∗*^	−0.014	0.001	0.044^*∗∗∗*^
	—	*(0.104)*	*(100.775)*	*(0.008)*	*(0.018)*	*(0.005)*	*(0.003)*	*(0.016)*	*(0.021)*	*(0.008)*	*(0.012)*	*(0.012)*

Locally unqualified providers	—	−0.841^*∗*^	912.782^*∗∗*^	−0.001	−	—	—	—	−0.357^*∗∗∗*^	—	0.466^*∗∗∗*^	—
	—	*(0.470)*	*(379.648)*	*(0.045)*	*−*	—	—	—	*(0.037)*	—	*(0.107)*	—
Mobile dental unit	—	0.182	—	0.231^*∗∗∗*^	0.332^*∗∗∗*^	—	—	−0.047	−0.309^*∗∗∗*^	—	—	—
	—	*(0.254)*	—	*(0.050)*	*(0.048)*	—	—	*(0.037)*	*(0.027)*	—	—	—

School	—	−0.539	—	0.777^*∗∗∗*^	−0.092	—	—	—	—	—	—	—
	—	*(0.669)*	—	*(0.111)*	*(0.093)*	—	—	—	—	—	—	—

Dental Insurance Coverage (Reference: SSS)												
Uninsured	0.034^*∗∗*^	0.410	2,103.760^*∗∗∗*^	0.027	−0.038	0.029	—	0.003	−0.032	0.022	0.011	0.013
	*(0.013)*	*(0.324)*	*(265.346)*	*(0.035)*	*(0.061)*	*(0.031)*	—	*(0.051)*	*(0.064)*	*(0.024)*	*(0.029)*	*(0.014)*

UCS	−0.036^*∗∗∗*^	0.170^*∗*^	−73.543	0.021^*∗∗*^	−0.111^*∗∗∗*^	−0.001	—	0.016	0.054^*∗∗∗*^	0.006	0.009	0.012^*∗*^
	*(0.003)*	*(0.099)*	*(99.638)*	*(0.009)*	*(0.019)*	*(0.002)*	—	*(0.016)*	*(0.019)*	*(0.004)*	*(0.009)*	*(0.006)*

CSMBS	−0.010^*∗∗∗*^	0.230^*∗∗*^	−1,222.615^*∗∗∗*^	0.015	−0.034^*∗*^	0.001	—	0.013	−0.018	0.013^*∗∗∗*^	0.012	0.016^*∗*^
	*(0.003)*	*(0.106)*	*(119.231)*	*(0.009)*	*(0.020)*	*(0.002)*	—	*(0.017)*	*(0.020)*	*(0.005)*	*(0.009)*	*(0.008)*

Private insurance	0.030^*∗∗*^	0.442	486.161^*∗∗*^	0.099^*∗∗*^	−0.180^*∗∗∗*^	—	—	−0.025	0.057	0.044∗	0.010	0.075^*∗∗*^
	*(0.012)*	*(0.278)*	*(244.514)*	*(0.040)*	*(0.041)*	—	—	*(0.040)*	*(0.055)*	*(0.025)*	*(0.026)*	*(0.033)*

Insurance paid by employer	0.148^*∗∗∗*^	0.287	−529.121^*∗*^	0.019	0.069	0.021	—	−0.081^*∗∗*^	−0.189^*∗∗∗*^	0.016	0.085	0.017
	*(0.025)*	*(0.288)*	*(290.715)*	*(0.029)*	*(0.058)*	*(0.022)*	—	*(0.032)*	*(0.062)*	*(0.019)*	*(0.053)*	*(0.016)*

Others insurances	0.014	0.078	−1,620.995^*∗∗∗*^	—	−0.053	—	—	0.060	0.099	—	—	—
	*(0.013)*	*(0.356)*	*(557.518)*	—	*(0.073)*	—	—	*(0.065)*	*(0.065)*	—	—	—

Insured but have to pay extra	—	0.988^*∗∗∗*^	2,107.128^*∗∗∗*^	0.010	−0.168^*∗∗∗*^	0.009^*∗∗*^	—	−0.027^*∗∗*^	0.048^*∗∗∗*^	0.043^*∗∗∗*^	0.055^*∗∗∗*^	0.093^*∗∗∗*^
	—	*(0.086)*	*(77.216)*	*(0.009)*	*(0.015)*	*(0.004)*	—	*(0.013)*	*(0.017)*	*(0.008)*	*(0.011)*	*(0.007)*

Constant	—	0.783^*∗∗∗*^	−1,101.953^*∗∗∗*^	—	—	—	—	—	—	—	—	—
	—	*(0.242)*	*(240.109)*	—	—	—	—	—	—	—	—	—

Observations	151,674	11,431	11,291	11,340	11,364	10,884	4,020	11,352	11,373	11,219	11,060	7,166
Pseudo R−squared	0.0441	0.0142	0.0565	0.102	0.146	0.102	0.134	0.0439	0.194	0.0923	0.219	0.418

Authors' Calculation. Source: Thailand's National Statistical Office. Italic numbers in parenthesis are standard error. ^*∗∗∗*^*p* < 0.01, ^*∗∗*^*p* < 0.05, ^*∗*^*p* < 0.1.

## Data Availability

Data are conducted from the national survey by Thailand's National Statistical Office.

## References

[1] Dave D., Kaestner R. (2009). Health insurance and ex ante moral hazard: evidence from Medicare. *International Journal of Health Care Finance and Economics*.

[2] Ehrlich I., Becker G. S. (1972). Market insurance, self-insurance, and self-protection. *Journal of Political Economy*.

[3] Arrow K. J. (1963). Uncertainty and the welfare economics of medical care. *The American Economic Review*.

[4] Taichman L. S., Sohn W., Lim S., Eklund S., Ismail A. (2009). Assessing patterns of restorative and preventive care among children enrolled in Medicaid, by type of dental care provider. *Journal of The American Dental Association*.

[5] Einav L., Finkelstein A. (2018). Moral hazard in health insurance: what we know and how we know it. *Journal of the European Economic Association*.

[6] Vujicic M., Buchmueller T., Klein R. (2016). Dental care presents the highest level of financial barriers, compared to other types of health care services. *Health Affairs*.

[7] Manning W. G., Bailit H. L., Benjamin B., Newhouse J. P. (1985). The demand for dental care: evidence from a randomized trial in health insurance. *Journal of The American Dental Association*.

[8] Srivastava P., Chen G., Harris A. (2015). Oral health, dental insurance and dental service use in Australia. *Health Economics*.

[9] Meyerhoefer C. D., Zuvekas S. H., Manski R. (2013). The demand for preventive and restorative dental services. *Health Economics*.

[10] Comptroller General’s Department (2016). The ministry of finance’s announcement of reimbursed medical expenses for public hospitals, B.E. 2559. *Comptroller General’s Department of The Ministry of Finance*.

[11] Social Security Office (2018). Criterion and compensation in the case of sickness or injury, which is irrelevant to work. Social security Office. https://www.sso.go.th/wpr/main/service/%E0%B8%81%E0%B8%AD%E0%B8%87%E0%B8%97%E0%B8%B8%E0%B8%99%E0%B8%9B%E0%B8%A3%E0%B8%B0%E0%B8%81%E0%B8%B1%E0%B8%99%E0%B8%AA%E0%B8%B1%E0%B8%87%E0%B8%84%E0%B8%A1_detail_detail_1_125_690/13_13.

[12] Petersen P. E., Baez R. J. (2013b). *Oral Health Surveys: Basic Methods*.

[13] Mejia G. C., Elani H. W., Harper S. (2018). Socioeconomic status, oral health and dental disease in Australia, Canada, New Zealand and the United States. *BMC Oral Health*.

[14] Bhatti T., Rana Z., Grootendorst P. (2007). Dental insurance, income and the use of dental care in Canada. *Journal*.

[15] Manski R. J., Macek M. D., Moeller J. F. (2002). Private dental coverage: who has it and how does it influence dental visits and expenditures?. *Journal of The American Dental Association*.

[16] Lee W., Kim S. J., Albert J. M., Nelson S. (2014). Community factors predicting dental care utilization among older adults. *Journal of The American Dental Association*.

[17] Wen P. C., Lee C. B., Chang Y. H., Ku L. J. E., Li C. Y. (2017). Demographic and rural–urban variations in dental service utilization in Taiwan. *Rural and Remote Health*.

[18] Minervini G., Russo D., Herford A. S. (2022). Teledentistry in the management of patients with dental and temporomandibular disorders. *BioMed Research International*.

[19] Caban-Martinez A. J., Lee D. J., Fleming L. E. (2007). Dental care access and unmet dental care needs among U.S. workers. *Journal of The American Dental Association*.

[20] Mueller C. D., Monheit A. C. (1988). Insurance coverage and the demand for dental care: results for non-aged white adults. *Journal of Health Economics*.

[21] Decker S. L., Lipton B. J. (2015). Do medicaid benefit expansions have teeth? the effect of medicaid adult dental coverage on the use of dental services and oral health. *Journal of Health Economics*.

[22] Elani H. W., Sommers B. D., Kawachi I. (2020). Changes in coverage and access to dental care five years after ACA Medicaid expansion. *Health Affairs*.

[23] Sweet M., Damiano P., Rivera E., Kuthy R., Heller K. (2005). A comparison of dental services received by Medicaid and privately insured adult populations. *Journal of The American Dental Association*.

[24] Cheema J., Sabbah W. (2016). Inequalities in preventive and restorative dental services in England, Wales and Northern Ireland. *British Dental Journal*.

[25] Taira K., Mori T., Ishimaru M. (2021). Regional inequality in dental care utilization in Japan: an ecological study using the national database of health insurance claims. *The Lancet Regional Health—Western Pacific*.

[26] Rezaei S., Woldemichael A., Zandian H., Homaie Rad E., Veisi N., Karami Matin B. (2018). Dental health-care service utilisation and its determinants in West Iran: a cross-sectional study. *International Dental Journal*.

[27] Bayat F., Akbarzadeh A., Monajemi F. (2017). Assessment of demand for and utilization of dental services by insurance coverage in a developing oral health care system. *Journal of Dental School*.

[28] Cornejo-Ovalle M., Paraje G., Vásquez-Lavín F., Pérez G., Palència L., Borrell C. (2015). Changes in socioeconomic inequalities in the use of dental care following major healthcare reform in Chile, 2004–2009. *International Journal of Environmental Research and Public Health*.

[29] NHSO (2017). Universal coverage scheme’s funding management handbook 2018. https://e-library.nhso.go.th/view/1/detail_ebook/28/TH-TH.

[30] NHSO (2020). Laws and order according to national health security act B.E. 2545- (A.D. 2002). https://e-library.nhso.go.th/view/1/detail_ebook/56/TH-TH.

[31] Potisomporn P., Sukarawan W., Sriarj W. (2019). Oral health education improved oral health knowledge, attitudes, and plaque scores in Thai third-grade students: a randomised clinical trial. *Oral Health and Preventive Dentistry*.

[32] Stasio D. D., Romano A., Paparella R. S. (2018). How social media meet patients’ questions: YouTube^™^ review for mouth sores in children. *Journal of Biological Regulators and Homeostatic Agents*.

[33] Laothong W., Cheng H. C. (2017). Comparison of factors affecting orthodontic treatment motivation of Taiwanese and Thai patients in two hospitals. *Journal of Dental Science*.

[34] Bazyar M., Yazdi-Feyzabadi V., Rashidian A., Behzadi A. (2021). The experiences of merging health insurance funds in South Korea, Turkey, Thailand, and Indonesia: a cross-country comparative study. *International Journal for Equity in Health*.

